# Supplementary material for: Chromothripsis during telomere crisis is independent of NHEJ, and consistent with a replicative origin
Source: Genome Res. 2019 May;29(5):737–49. doi: 10.1101/gr.240705.118 (PMC6499312; doi:10.1101/gr.240705.118)
Supplement: Supplemental Material [file supp_gr.240705.118_Supplemental_file_1.zip › contigs/annotated_contigs/DB49/contig.3.DB49_length_750_mean_cov_8.95466666667.docx]

**DB49_length_750_mean_cov_8.95466666667**

```
AAAAAAGTCTGATACTTAAAAAAA|ACATAAAGCAAAAACACTGCACAGACCATGGGGTTGATAGGCTCAAAGCATCATGTGGTATAAA
                         >chr12:48233266-48233593 - E=7e-185
TAGCTCACTGGTGTGCTAGGAGTATTGATTCCTTTAGCCCTGGAGCAAGCAAACAGGGCCTGCCAGGAGTGACCACAGCCCTTCAATTT

CCCCAGCTTCTACCAGGCTCCTTGCAGGCTGCCTGTGCAGTGCAGGTCGGTCTGCCTGCCCCATGGTCCCTGCAGATGACAAGAAGGAT

GGATGCTGTCTGACACCTCCAGCATGGCCAAGGAGATGGCTCATCATGCTGACATCCTATAGGCAACTAGTCCTCATTGTGGGC|A|TA
                                                                                     >c
ACAACATTTGGCCAGCTTTCTCTTTGTTTCCACTGGACACAAGCTCACCTACCAGCGACAGGAGGGGGACAATGCTCCCGCGCCTCCTC
hr12:48410984-48411361 - E=5e-215
CCTCTTTCTCCCCTGGAACTGTGCTCAGACCTAGTCAGGGGACAGGGAGAGCCTCATTCACATGTGGCTTATGGCCCAGCGACCTGCTG

GCTAACAAAGCTTTCTAAGGATATCTGAGAGAAGCTTGGTACCCCATAGGAGGAAGGGACAGCAACAGAGGTGCCCTGCCCGGTATCCT

GGGACCCAACCAGTGCTGCCCTCTCACCTCATCCCAAGATGGGCAAAGCAGCACAGCTTGCTCTCTCGTAACCTTCCTGAGGGCACCAT

TGTCTTGACCGAGT|CCTC|AGGAAGGTTACGAGAGAGCAAGC               >chr12:48411009-48411036 +
```
